# Isolation, Characterization and Whole Genome Analysis of an Avian Pathogenic *Escherichia coli* Phage vB_EcoS_GN06

**DOI:** 10.3390/vetsci9120675

**Published:** 2022-12-05

**Authors:** Leping Wang, Yizhou Tan, Yuying Liao, Lei Li, Kaiou Han, Huili Bai, Yajie Cao, Jun Li, Yu Gong, Xiaoye Wang, Hao Peng

**Affiliations:** 1 College of Animal Science and Technology, Guangxi University, Nanning 530004, China; 2 Guangxi Zhuang Autonomous Region Engineering Research Center of Veterinary Biologics, Nanning 530004, China; 3 Guangxi Key Laboratory of Veterinary Biotechnology, Guangxi Veterinary Research Institute, Nanning 530001, China; 4 Animal Science and Technology Station of Guizhou, Guiyang 550000, China

**Keywords:** phage, avian pathogenic *Escherichia coli*, whole genome analysis

## Abstract

**Simple Summary:**

In this study, an avian pathogenic *Escherichia coli* phage, named vB_EcoS_GN06, was isolated from sewage. Characterization experiments demonstrated that phage GN06 had wide tolerances of pH and temperature, and the one-step growth experiments showed that the latent time is 60 min, the burst size is 434 PFU/cell, and that phage GN06 has a strong lytic ability and significantly inhibits host in vitro growth and biofilm formation of the bacterium. Based on the whole genome analysis, phage GN06 can be assigned to the genus *Tequintavirus* and it is safe in the application. In summary, phage GN06 infecting avian pathogenic *Escherichia coli* GXEC-N22 (O78), may provide alternative materials for future phage therapy.

**Abstract:**

*Escherichia coli* (O78) is an avian pathogenic *Escherichia coli* (APEC). It can cause perihepatitis, pericarditis, septicemia and even systemic infections in the poultry industry. With the incidence of antibiotic resistance reaching a crisis point, it is important to find alternative treatments for multidrug-resistant infections. The use of phages to control pathogens is a promising therapeutic option for antibiotic replacement. In this study, we isolated a lytic phage called vB_EcoS_GN06 from sewage. It lysed APEC GXEC-N22. Transmission electron microscopy showed that the phage belongs to family *Siphoviridae*. Phage GN06 has a 107,237 bp linear double-stranded DNA genome with 39.2% GC content and 155 coding sequences. It belongs to the genus *Tequintavirus*, subfamily *Markadamsvirinae*. The multiplicity of infection of 0.01 and the one-step growth showed that the latent time is 60 min and the burst size is 434 PFU/cell. Temperature and pH stability tests showed that phage GN06 was stable in the range of 4 °C–60 °C and pH 5–9. GN06 showed significant inhibition of APEC both within the liquid medium and in biofilm formation. These results suggest that phage GN06 has the potential to control bacterial pathogens. Thus, GN06 has the potential to be a new potential candidate for phage therapy.

## 1. Introduction

Avian pathogenic *Escherichia coli* (APEC) can cause avian colibacillosis, leading to systemic infections and perihepatitis, pericarditis, and septicemia, causing great losses to the poultry industry [1]. In addition, APEC is a reservoir of drug resistance and virulence genes for human extraintestinal pathogenic *E. coli*, which is a potential threat to human health and public health. Currently, antibiotics are mainly relied on to combat APEC infections [2]. However, the widespread and improper use of antibiotics has led to the emergence of drug-resistant *E. coli* strains, even multiple drug-resistant *E. coli* multidrug-resistant bacteria (MDR) or extensively drug-resistant (XDR) *E. coli* [3]. In 2017, methicillin-resistant and MDR *E. coli* were listed as antibiotic-resistant priority pathogens by WHO [4]. In addition, biofilm can form during APEC infection, which in turn leads to antibiotic resistance and persistent infection [5,6]. Therefore, there is an urgent need to develop alternative methods of antibiotics to prevent and control bacterial infections.

Recently, the phage has been considered to be one of the effective methods against the multiple drug resistance of bacteria [7]. Phage has the characteristics of wide distribution, variety, and strong host specificity, so it can be used as a new biological agent for the treatment of bacterial infection [8]. Unlike antibiotics, phages can proliferate rapidly and eliminate host bacteria. And they will not cause substantial damage to the normal flora of animals or humans [9]. Many studies have shown that phages can effectively treat experimentally infected animals, and phages have become one of the most promising measures against drug-resistant bacteria [10,11,12]. Moreover, phages have been commercialized to deal with the current problems of food safety, environmental disinfection, and the treatment of bacterial diseases [13,14]. Many phages of *E. coli* have been isolated and sequenced, but few phages are suitable for clinical application, including the development of phage resistance, regulation of phage-based products, and unknown environmental consequences of phage treatment [15]. Therefore, it is necessary to continue to search for natural *E. coli* phages.

In this study, we used APEC GXEC-N22 as the host bacterium and isolated an *Escherichia* phage named vB_EcoS_GN06 (hereinafter referred to as GN06) from the sewage of an avian farm in Guangxi. Based on the results of biological characteristics and whole genome analysis, phage GN06 was assumed to have the potential of being a biocontrol agent against multidrug-resistant *Escherichia coli* strains. Furthermore, the whole genomic analysis of phage GN06 will provide useful information for the further study of interactions between bacteria and phages.

## 2. Materials and Methods

### 2.1. Isolation and Purification of Phage and Electron Microscopic Observation

Sewage recovered from the pig farm treatment station in Guangxi was centrifuged at 5000× *g* for 30 min at 4 °C, before the supernatant was removed and filtered through a 0.22 µm microporous membrane. A 100 µL aliquot of the above filtrate was added to a 5mL culture of log period *E. coli* GXEC-N22 and incubated overnight at 37 °C and 220 rpm. The *E. coli* was then centrifuged at 5000× *g* for 2 min and the supernatant was collected by filtering through a 0.22 µm microporous membrane. Phage purification was carried out by three continuous single-plaque isolations using the double-layered agar method and phage stock was stored at 4 °C until use in further experiments [16].

After the filtered phage performed a ten-fold serial dilution, 100 μL phage liquid was mixed with 100 μL *E. coli* GXEC-N22 and, using the method of double layer tablets after observing plaque growth situation at 37 °C for 12 h, determination of phage titer was made by counting the number of plaques. High titer of purified phage (10^10^ PFU/mL) was deposited on copper grids with carbon-coated Formvar films (Beijing Zhongjingkeyi Technology Co., Ltd., Beijing, China) and stained with Phosphotungstic acid (PTA, 2% w/v) for 5–10 min and dried for 30 min. The morphology of phage GN06 was examined using a HITACHI HT-7700 transmission electron microscope (TEM, Hitachi High-Tech Co., Ltd., Tokyo, Japan). 

### 2.2. Determination of Phage Lysis Profile

In this study, a total of 56 *E. coli* strains were tested. Among these, twenty-three strains were isolated from avian farms, four strains were isolated from hospitals in Guangxi, fifteen strains were isolated from a slaughterhouse in Guangdong, ten strains were isolated from avian farms in Sichuan, two strains were isolated from a pet hospital in Guangxi and two strains were purchased from China Veterinary Culture Collection Center (CVCC) (Table 1). Each strain of *E. coli* comes from a different place and was saved in the clinical veterinary laboratory of Guangxi University. These strains were resuscitated at 37 °C using LB broth.

### 2.3. Optimal Multiplicity of Infection and One-Step Growth Curve

For optimal multiplicity of infection (MOI), phage suspension (100 µL) and equal volume of GXEC-N22 were mixed at MOIs of 0.001, 0.01, 0.1, 1, 10, 100 and aliquots were taken after incubation for 5 h, and then phage titer were measured by the double-layer agar method. 

One-step growth experiments were performed by a modification of methods described previously [17]. The logarithmic phase was mixed with GXEC-N22 (MOI = 0.01), 37 °C warm bath after 15 min 5000 rpm centrifugal for 1 min, the supernatant discarded, and then the sample was washed with LB to precipitate. Then, the mixture was suspended in preheated LB broth, followed by incubation at 37 °C. Samples were taken at 10 min intervals up to 90 min and immediately diluted, and then phage titers were determined by the method mentioned above. The calculation formula of burst size was: Burst size = phage titer at the end of lysis/number of host strains at the beginning of infection. All tests were repeated three times.

### 2.4. Thermolability and pH Sensitivity

For thermostability testing, samples of the phage suspension were incubated at 4, 25, 37, 50, 60, 70, and 80 °C, and aliquots were taken after incubation for 30, 60, and 90 min. Then phage titer was determined by methods of the double-layer agar method. For pH stability testing, samples of the isolated phage were mixed in a series of tubes containing TM (50 mM Tris, 10 mM MgSO_4_) of different pH values (2–12, adjusted using NaOH or HCl), incubated for 2 h at 37 °C, and then titered by the double-layer agar plate method.

### 2.5. In Vitro Inhibition Test of Phage

The in vitro inhibition assay of the phage was performed with reference to the previously described method [18]. The host bacterium GXEC-N22 was incubated to about OD_600_ = 0.2 (Approximately 10^8^ CFU/mL), and the phage was added at MOI = 0.01, 0.1 and 1, respectively. An equal volume of GXEC-N22 treated with PBS buffer was used as control and a medium control group with only LB medium added was set up. Bacterial growth was determined by measuring OD_600_ every 2 h.

### 2.6. Determination of the Ability of Phages to Inhibit the Biofilm of Escherichia coli

The ability of phages to inhibit the formation of *E. coli* biofilm was examined by the crystalline violet staining method in the literature [19]. The host bacteria GXEC-N22 and phage GN06 were inoculated in 96-well plates at MOI = 0.01, 0.1, 1, respectively, and incubated at 37 °C for 24 h. A medium control group with only LB medium added was set up. The plates were washed twice with PBS, dried naturally, and stained with 2 g/L crystal violet for 20 min. The biofilms were washed twice with PBS, dried well, and dissolved in 950 mL/L ethanol for the determination of OD_595_.

### 2.7. Extraction and Analysis of Phage vB_EcoS_GN06 Genome

Genomic DNA of phage GN06 was extracted from a high titer (10^10^ PFU/mL) of purified phages using the methods described above and extracted by an alkaline lysis method. After the phage pellet was suspended in SM (5.8 g of NaCl, 2.0 g of MgSO_4_ · 7H_2_O, 50 mL of Tris-HCl [Ph 7.4], 5.0 ml of 2% gelatin) buffer, sodium dodecyl sulfate (SDS) (final concentration, 0.5%) and proteinase K (final concentration, 50 μg/mL) were added. In order to remove residual bacterial DNA, DNase I (500 μg/mL) and RNase A (100 μg/mL) were added to the mixture and incubated at 37 °C for 30 min. The mixture was vortexed thoroughly and incubated at 56 °C overnight. An equal volume of phenol-chloroform was added to remove the proteinaceous material. The extraction was repeated twice, and phage DNA was precipitated according to ethanol precipitation procedures. The pellet was dissolved in 30 μL of buffer TE, and the isolated nucleic acids were separated using 0.8% agarose gel electrophoresis, stained with ethidium bromide, and analyzed under ultraviolet (UV) light. After digestion, electrophoresis of the samples in 0.8% agarose containing ethidium bromide (1 μg/mL) was performed. The method of sequencing adopted whole genome shotgun (WGS). The sequencing platform was Illumina Miseq, sequencing mode was paired-end (2 × 250 bp) and library insert size was 400 bp.

The complete genome sequence was annotated using Subsystem Technology (RAST, http://rast.nmpdr.org, accessed on 21 August 2022) and GeneMark (http://opal.biology.gatech.edu/GeneMark/, accessed on 23 August 2022) [20,21]. All predicted open reading frames (ORFs) were verified using the online BLASTP (http://www.ncbi.nlm.nih.gov/BLAST, accessed on 2 September 2022). The putative transfer RNA (tRNA)-encoding genes were searched using tRNA scan-SE (http://trna.ucsc.edu/tRNAscan-SE/, accessed on 15 October 2022). The tools of genome visualization chose The CGView Server (CGView Server) and Easyfig_2.2.5_win [22]. Easyfig is creating linear comparison figures of multiple genomic loci based on BLAST. In this study, four sequences (*Escherichia* phage vB_EcoS_HASG4, *Escherichia* phage vB_EcoS_AKFV33, *Yersinia* phage phiR201, *Enterobacteria* phage EPS7) joined in the creating linear comparison. The virulence genes and acquired drug resistance genes test was used VirulenceFinder 2.0 and ResFinder 4.1 [23]. The phylogenetic tree of phage GN06 was constructed based on the large terminator subunit using the ClustalW program in MEGA7 [24].

### 2.8. Statistical Analysis

All statistics were analyzed using GraphPad Prism 9.0 (GraphPad Software, Inc., San Diego, CA, USA). The significance of experimental data was assessed with multiple *t*-tests. The error bars represent the standard deviation (SD) of the mean.

## 3. Results

### 3.1. Characteristics of Phage vB_EcoS_GN06

**Figure 1:**
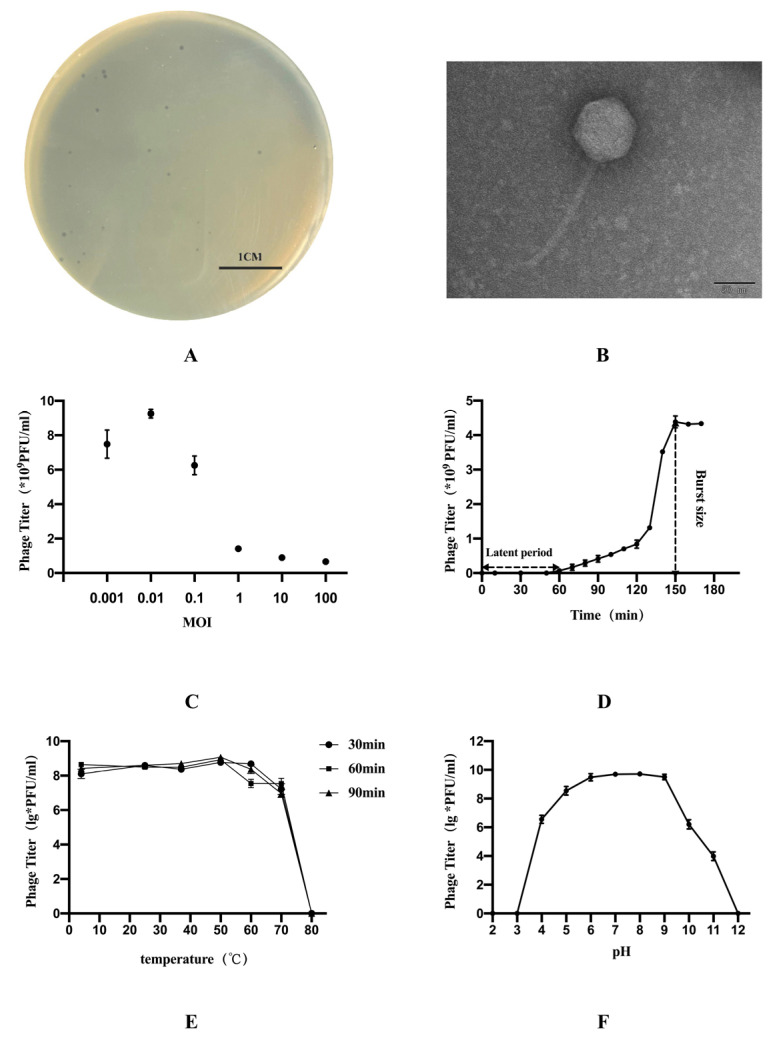
The morphology, biological characteristics of phage vB_EcoS_GN06: (**A**) Plaques of phage GN06. (**B**) Transmission electron microscopy (TEM) of phage GN06. (**C**) Determination of optimal multiplicity of infection (MOI) of phage GN06. (**D**) One-step growth curve of phage GN06. (**E**) Thermal stability of GN06. (**F**) Stability of phage GN06 in different pH values.

The results of morphology on the double-layer agar plate showed that the plaque was clear with 1 mm in diameter (Figure 1A). The TEM image showed that phage GN06 had a polyhedral head (84 nm in diameter) and a long tail (167 nm in length) (Figure 1B). Phage GN06 is the highly specific and only lyses host bacteria GXEC-N22 (Table 1). 

The highest titer of phage GN06 was released under the multiplicity of infection of 0.01. One-step growth experiments were performed to assess the lytic capability of phage GN06 with an MOI of 0.01 (Figure 1C). The one-step growth experiments showed that the latent time is 60 min and the burst size is 434 PFU/cell (Figure 1D). 

The thermal stability test showed that phage GN06 was stable at 4–60 °C. However, phage GN06 was completely inactivated at 80 °C (Figure 1E). In addition, phage GN06 remained high activity over a broad pH range of pH 5–9 for 2 h, but it was inactivated when exposed to strong alkali or acid (pH < 4 or pH > 12) (Figure 1F).

### 3.2. In Vitro Phage vB_EcoS_GN06 Inhibition Test

**Figure 2:**
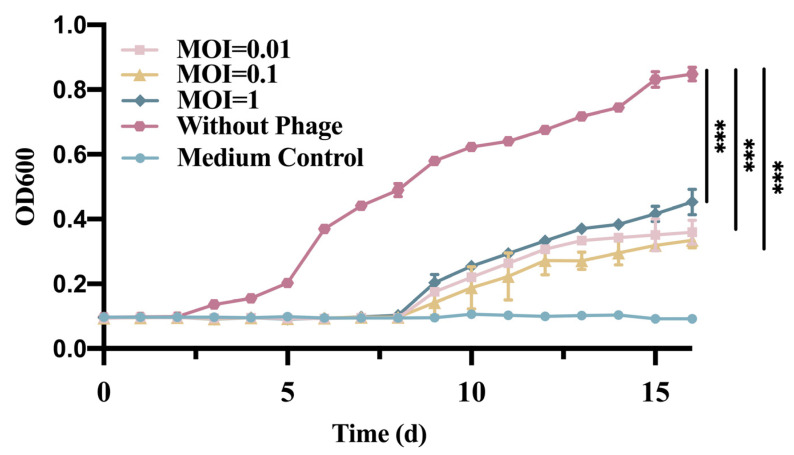
Effect of phage vB_EcoS_GN06 treatments on the growth of *E. coli* GXEC-N22 at different MOI in MH broth (***: *p* < 0.001).

The control group without phage continued to grow during the 12-h incubation; Phage GN06 had a significant inhibitory effect on the growth of *E. coli* GXEC-N22 when the MOI was 0.01, 0.1 and 1, respectively, when phage was added to the culture medium (Figure 2).

### 3.3. Determination of the Ability of Phage vB_EcoS_GN06 to Inhibit the Biofilm of Escherichia coli

**Figure 3:**
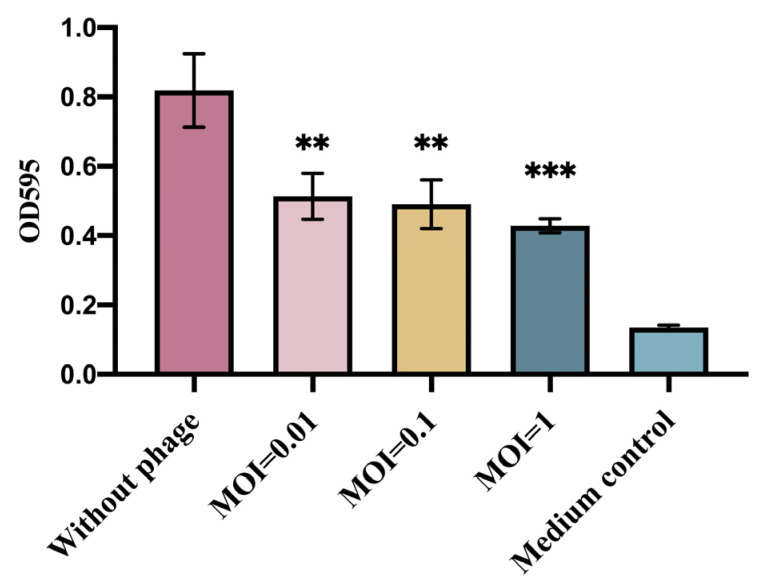
Inhibition of bacterial biofilm formation by phage vB_EcoS_GN06. The columns in the figure represent Control (without phage), The experimental groups added with phage at MOI = 1, 0.1 and 0.01, and medium control, respectively (**: *p* < 0.01, ***: *p* < 0.001).

The inhibition of *E. coli* biofilm formation by phage GN06 was examined by 96-well plate crystalline violet staining. The phage significantly inhibited the biofilm formation of GXEC-N22 at MOI = 0.01, 0.1 and 1 compared to the negative control (Figure 3) (*p* < 0.01, *p* < 0.001).

### 3.4. The Whole Genomic Analysis of the Phage vB_EcoS_GN06

**Figure 4:**
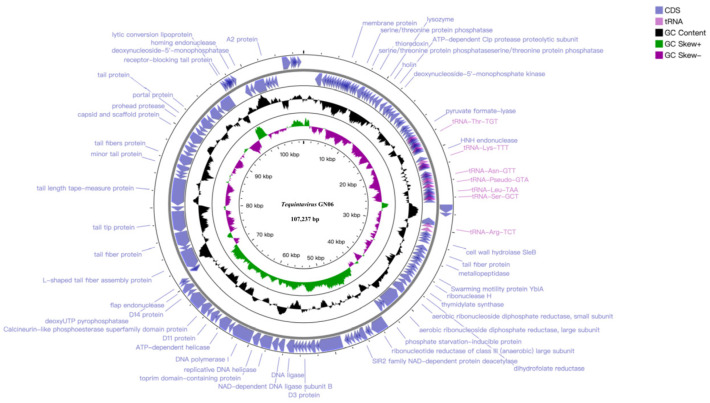
Genome map of phage vB_EcoS_GN06, the genome of phage GN06 depicted in the circular. These arrows represent 155 CDS, among which 32 CDS can be predicted to function. In addition, the map shows GC skew and content about the genome. Note: Not marked as hypothetical protein.

The whole genomic analysis indicated that phage GN06 has double-stranded DNA of length 107, 237 bp, with a G+C% content of 39.20% (Figure 4). BLASTn analysis showed that phage GN06 shares 94.07% identity (88% genome coverage) with *Escherichia* phage vB_EcoS_HASG4 (GenBank accession MK373797.1). The genome contained 155 predicted open reading frames (CDSs), and the inverted CDSs accounted for 69.03% (107 CDSs) of the total genome. No virulence genes, drug-resistance genes or lysogeny genes (e.g., integrase) were detected. Among the one hundred and fifty-five CDSs, of which fifty had been assigned to functional genes; four CDSs encode proteins related to DNA replication and repair; ten CDSs are involved in encoding phage structure proteins; four CDSs are associated with phage cleavage and thirty-two CDSs are responsible for encoding proteins related to transcriptional regulation. The remaining 104 were annotated as hypothetical proteins and 1 no-hits protein (Supplementary Material). In addition, nineteen tRNA genes were found in the genome of phage GN06 and two of them were predicted to be pseudogenes. The annotated complete genome sequence of the GN06 phage was deposited in GenBank under accession number OM867526.

### 3.5. Comparative Genomics and Phylogenetic Analysis of Phage vB_EcoS_GN06

**Figure 5:**
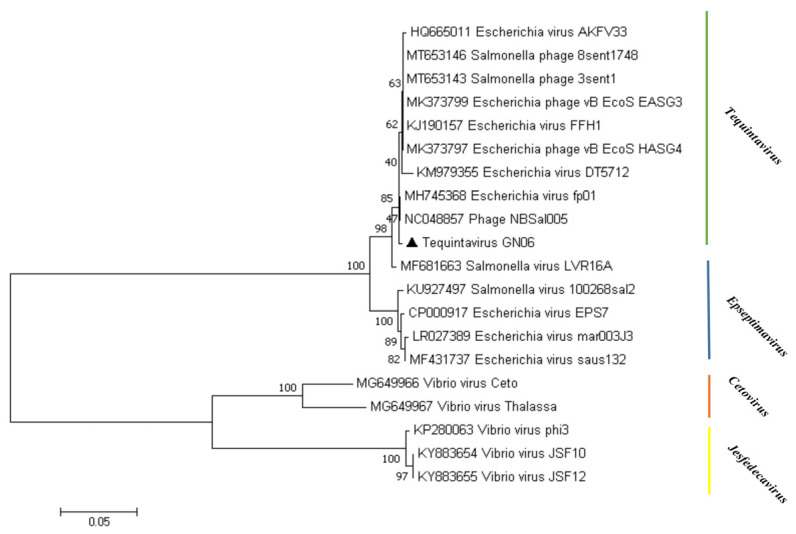
Phylogenetic analysis of phage vB_EcoS_GN06. The phylogenetic tree was constructed using the neighbor-joining method with 1000 bootstrap replicates. Amino acid sequences of the terminase large subunit of phage GN06 and other phages within the subfamily *Markadamsvirinae* were aligned in the MEGA7.

To investigate the evolutionary relationship that phage GN06 from the “*Tequintavirus*” displays within the *Markadamsvirinae* subfamily, phylogenetic analyses were performed on the whole genome of 20 published phage genomes and GN06 (Figure 5). In the phylogram tree the phage GN06 was more closely related to “*Tequintavirus*” (phage NBSal005 and fp01). In addition, the “*Tequintavirus*” cluster into a single monophyletic clade.

**Figure 6:**
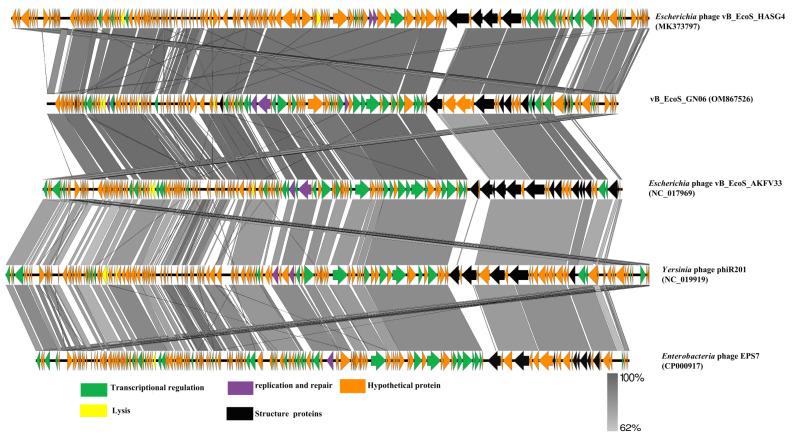
Comparative analysis of five phages was conducted using Easyfig, with nucleotide identity above 62%. Well-conserved segments were paired by shaded regions, and arrows indicated the direction of transcription for the predicted CDSs.

For further similarity analysis, the phage GN06 was subjected to genomic multiple comparisons with the phage of the genus “*Tequintavirus*”. The results showed that phage GN06 had a high protein homology with them (>62%). The homologous proteins modules mainly include DNA transcriptional regulation, DNA replication and repair, lysis, and structural protein, among others (Figure 6).

## 4. Discussion

APEC infection of birds causes avian *E. coli* disease, which brings great losses to the poultry industry, with complex and diverse serotypes and no cross-protection, making prevention and control difficult [25]. Antibiotics are mainly used clinically to prevent and control avian *E. coli* disease; however, the large incidence of irrational use of antibiotics has led to the widespread emergence of multi-drug resistant APEC strains, which has caused a great obstacle to disease prevention and control [26]. Secondly, antibiotic abuse causes drug residues and imbalances in the breeding environment flora, which seriously threaten human health. In response to the problem of antibiotics, the relevant departments have introduced a series of anti-ban policies to limit antibiotics [27]. At the same time, there is an urgent need to develop alternatives to antibiotics to prevent and control drug-resistant bacterial infections. Phage is a promising alternative to antibiotics, which has the advantages of high specificity, high safety and low resistance compared with antibiotics, and which has received wide attention from scholars at home and abroad [28].

In this study, an *E. coli* phage vB_EcoS_GN06 was isolated from the farm sewage of a poultry farm in Guangxi, and it was observed by transmission electron microscopy to have a tail size of 167 nm and belong to the family *Siphoviridae*. Whole genome sequencing and phylogenetic analysis showed that phage GN06 belongs to the subfamily *Markadamsvirinae* and genus *Tequintavirus*. Phage GN06 survived stably in the temperature range of 4–70 °C, and possessed a higher temperature tolerance range compared to Li et al. study [29]. The phage remained active at pH 4–11 and possessed a higher tolerance range for acidity compared to Zhang et al. [30], but when applying phage orally for the treatment of enterotoxic or enteropathogenic *E. coli*, the high acidity of gastric juice (pH < 3) may affect phage potency and thus reduce therapeutic efficacy. Colom’s study found that phage could be combined with sodium alginate into a microcapsule that can significantly enhance the pH tolerance of the phage [31], which also provides a good idea for the future application of phage GN06. The one-step growth curve reflects the latency and burst size of the phage, which is one of the most valuable indicators to evaluate the potential of phage application. The shorter incubation period and larger burst size of phage GN06 indicate that the phage infects and increases rapidly, while phages with shorter incubation time are able to lyse more bacteria within a certain period of time and are more suitable for biocontrol applications [32]. APEC can form biofilms on living or non-living surfaces. The biofilm consists of extracellular polysaccharides, proteins, and DNA, which can help bacteria increase their resistance to external environmental stresses, leading to antibiotic resistance and persistent infection, making treatment more difficult [33]. We found that vB_EcoS_GN06 was effective in inhibiting the growth and biofilm formation of host bacteria at MOIs of 0.01, 0.1 and 1, respectively, and its strong lytic ability indicated its potential to lyse bacterial infections.

In addition, phages use their receptor-binding proteins (RBPs) to bind to specific receptors on the surface of the bacterium. These include tail and tail spike proteins. Because of the high specificity of RBPs binding to antigens, phages encode different RBPs to match the diversity of O antigens [34]. These RBPs enable phages to bind to different O antigens of lipopolysaccharide (LPS), allowing them to recognize different receptors [35]. The phage GN06 genome has 3 CDSs responsible for coding RBPs. Compared with Zhou et al. [36], GN06 had fewer RBPs, which may be one of the important reasons for the ability of phage GN06 to lyse only one serotypes of *E. coli*.

Identification of biological characteristics and genome sequencing permits the rapid identification of undesirable features to quickly eliminate unsuitable phages [37]. The phage described in this work was characterized by a number of favorable features, and the absence in its genome of genes encoding potential toxins was noted. These characteristics were evidence for phage GN06 to have reliable performance in an in vivo environment. Phage infections cause dramatic changes in the intracellular environment of host bacteria, which ensure the specific and rapid macromolecular synthesis necessary for phage development [38]. Phages have highly compact genomes, and the genome size reflects their capacity to utilize the host’s resources. The tRNAs are only translation-associated genes usually found in phages, particularly those with large genomes [39]. Nineteen tRNA genes, including two pseudogenes, were discovered in the genome of phage GN06, there is a significant association between tRNA distribution and codon usage; the tRNAs in the genomes of virulent phages tend to correspond to highly used codons, leading to an enhanced translational efficiency [40]. Though most phages contain only one or two tRNAs, a few phages contain more than 20 such sequences, nearly as many as tRNAs in bacteria with minimal genomes. All phages containing tRNA are dsDNA phages. The phages with tRNAs differ from those without tRNAs in genome length. Compared to temperate phages, virulent phages contain more tRNAs, more codon usage biases, and more compositional differences relative to the host genome [41]. In summary, the phage GN06 was inferred to be among the virulent phages. The same result was obtained in our in vitro tests.

## 5. Conclusions

In this study, an avian pathogenic *Escherichia coli* phage, named phage vB_EcoS_GN06, was isolated from sewage. Characterization experiments demonstrated that phage GN06 had wide tolerances of pH and temperature and the one-step growth experiments showed that its latent time is 60 min and its burst size is 434 PFU/cell, while GN06 has a strong lytic ability and significantly inhibits host in vitro growth and biofilm formation of the bacterium. Based on the whole genome analysis, phage GN06 can be assigned to the genus *Tequintavirus* and it is safe in the application. In summary, phage GN06, a phage infecting avian pathogenic *Escherichia coli* GXEC-N22 (O78), may provide alternative materials for future phage therapies.

## Figures and Tables

**Table 1:** Bacterial strains information.

Strains	Genus	Place	Source	Serotype/Capsular Type	Sensitivity
GXEC-N01	*Escherichia coli*	Guangxi	Avian	O157: H7	-
GXEC-N02	*Escherichia coli*	Guangxi	Human	Undetected	-
GXEC-N03	*Escherichia coli*	Guangxi	Human	Undetected	-
GXEC-N04	*Escherichia coli*	Guangxi	Avian	O126: K71(B16)	-
GXEC-N05	*Escherichia coli*	Guangxi	Avian	O26: K60(B6)	-
GXEC-N06	*Escherichia coli*	Guangxi	Avian	O126: K71(B16)	-
GXEC-N07	*Escherichia coli*	Guangxi	Avian	O157: H7	-
GXEC-N08	*Escherichia coli*	Guangxi	Avian	Undetected	-
GXEC-N09	*Escherichia coli*	Guangxi	Avian	Undetected	-
GXEC-N10	*Escherichia coli*	Guangxi	Avian	Undetected	-
GXEC-N11	*Escherichia coli*	Guangxi	Avian	O142: K86(B)	-
GXEC-N12	*Escherichia coli*	Guangxi	Human	O127a: K63(B8)	-
GXEC-N13	*Escherichia coli*	Guangxi	Avian	Undetected	-
GXEC-N14	*Escherichia coli*	Guangxi	Avian	Undetected	-
GXEC-N15	*Escherichia coli*	Guangxi	Avian	Undetected	-
GXEC-N16	*Escherichia coli*	Guangxi	Avian	Undetected	-
GXEC-N17	*Escherichia coli*	Guangxi	Avian	Undetected	-
GXEC-N18	*Escherichia coli*	Guangxi	Avian	Undetected	-
GXEC-N19	*Escherichia coli*	Guangxi	Avian	Undetected	-
GXEC-N20	*Escherichia coli*	Guangxi	Avian	Undetected	-
GXEC-N21	*Escherichia coli*	Guangxi	Avian	Undetected	-
GXEC-N22	*Escherichia coli*	Guangxi	Avian	O78	+
GXEC-N23	*Escherichia coli*	Guangxi	Avian	Undetected	-
GXEC-N24	*Escherichia coli*	Guangxi	Avian	Undetected	-
GXEC-N25	*Escherichia coli*	Guangxi	Avian	Undetected	-
GXEC-N26	*Escherichia coli*	Guangxi	Avian	Undetected	-
GXEC-N27	*Escherichia coli*	Guangxi	Human	Undetected	-
GXEC-B01	*Escherichia coli*	Guangxi	Pet	Undetected	-
GXEC-B02	*Escherichia coli*	Guangxi	Pet	Undetected	-
GXEC-C01	*Escherichia coli*	Guangxi	Bovine	O127a: K63(B8)	-
GDEC-F01	*Escherichia coli*	Guangdong	Pig	Undetected	-
GDEC-F02	*Escherichia coli*	Guangdong	Pig	Undetected	-
GDEC-F03	*Escherichia coli*	Guangdong	Environment	Undetected	-
GDEC-F04	*Escherichia coli*	Guangdong	Environment	O127a: K63(B8)	-
GDEC-F05	*Escherichia coli*	Guangdong	Environment	O127a: K63(B8)	-
GDEC-F06	*Escherichia coli*	Guangdong	Environment	O127a: K63(B8)	-
GDEC-F07	*Escherichia coli*	Guangdong	Environment	O111: K58(B4)	-
GDEC-F08	*Escherichia coli*	Guangdong	Environment	Undetected	-
GDEC-F09	*Escherichia coli*	Guangdong	Environment	Undetected	-
GDEC-F10	*Escherichia coli*	Guangdong	Environment	Undetected	-
GDEC-F11	*Escherichia coli*	Guangdong	Avian	Undetected	-
GDEC-F12	*Escherichia coli*	Guangdong	Avian	Undetected	-
GDEC-F13	*Escherichia coli*	Guangdong	Avian	Undetected	-
GDEC-F14	*Escherichia coli*	Guangdong	Avian	Undetected	-
GDEC-F15	*Escherichia coli*	Guangdong	Environment	Undetected	-
SCEC-Z01	*Escherichia coli*	Sichuan	Avian	Undetected	-
SCEC-Z02	*Escherichia coli*	Sichuan	Avian	Undetected	-
SCEC-Z03	*Escherichia coli*	Sichuan	Avian	Undetected	-
SCEC-Z04	*Escherichia coli*	Sichuan	Avian	Undetected	-
SCEC-Z05	*Escherichia coli*	Sichuan	Avian	Undetected	-
SCEC-Z06	*Escherichia coli*	Sichuan	Avian	Undetected	-
SCEC-Z07	*Escherichia coli*	Sichuan	Avian	O157	-
SCEC-Z08	*Escherichia coli*	Sichuan	Avian	Undetected	-
SCEC-Z09	*Escherichia coli*	Sichuan	Avian	Undetected	-
SCEC-Z10	*Escherichia coli*	Sichuan	Avian	Undetected	-
CVCC1527	*Escherichia coli*	China Veterinary Culture Collection Center (CVCC)	Pig	O8: K88	-
CVCC4050	*Escherichia coli*	China Veterinary Culture Collection Center (CVCC)		O157: H7	-

“+”: Lytic; “-”: Not lytic. All bacteria were isolated from different farms.

## Data Availability

All data and material are available upon request to the corresponding author.

## Supplementary Materials

The following supporting information can be downloaded at: https://www.mdpi.com/article/10.3390/vetsci9120675/s1, Table S1. ORFs description.
