# Supplementary material for: Isolation, Characterization and Whole Genome Analysis of an Avian Pathogenic Escherichia coli Phage vB_EcoS_GN06
Source: Vet Sci. 2022 Dec 5;9(12):675. doi: 10.3390/vetsci9120675 (PMC9788193; doi:10.3390/vetsci9120675)
Supplement: Supplementary file 1 [file vetsci-09-00675-s001.zip › vetsci-2042351-supplementary.pdf]

Table S1. ORFs description.

CDS	start	stop	identity	length（aa）	function	e-value	accession number
1	1537	2427	99.32%	296	hypothetical protein [Salmonella phage SP76]	0.0	QPI16080.1
2	2427	3041	100.00%	61	hypothetical protein [Escherichia phage vB_EcoS_AKFV33]	1.E-30	YP_006382319.1
3	3041	3226	99.32%	146	hypothetical protein [Salmonella enterica subsp. enterica serovar Derby]	5.E-106	ECJ4201711.1
4	3226	3666	98.04%	204	hypothetical protein [Salmonella enterica subsp. enterica serovar Bareilly]	4.E-149	YP_004306501.1
5	3668	3871	100.00%	67	hypothetical protein SPC35_0022 [Salmonella virus SPC35]	2.E-41	YP_004306505.1
6	3926	4264	99.11%	112	hypothetical protein HOT55_gp027 [Salmonella phage SP3]	1.E-72	YP_009804458.1
7	4245	4706	99.35%	153	hypothetical protein A318_gp026 [Escherichia phage vB_EcoS_AKFV33]	4.E-110	YP_006382325.1
8	4703	4903	98.48%	66	hypothetical protein SPC35_0025 [Salmonella virus SPC35]	5.E-38	YP_004306508.1
9	5003	5329	99.07%	108	hypothetical protein [Salmonella phage vB_SalS_ABTNLsp4]	1.E-71	QPI13133.1
10	5319	5564	100.00%	81	putative membrane protein [Salmonella phage bux]	7.E-50	QIQ61667.1
11	5561	5842	100.00%	93	hypothetical protein A318_gp030 [Escherichia phage vB_EcoS_AKFV33]	1.E-56	YP_006382329.1
12	5842	6003	96.23%	53	hypothetical protein SPC35_0029 [Salmonella virus SPC35]	2.E-27	YP_004306512.1
13	6003	6254	100.00%	83	hypothetical protein SPC35_0030 [Salmonella virus SPC35]	1.E-51	YP_004306513.1
14	6333	6764	100.00%	143	hypothetical protein A318_gp032 [Escherichia phage vB_EcoS_AKFV33]	9.E-101	YP_006382331.1
15	6817	6939	97.50%	40	hypothetical protein HOT55_gp036 [Salmonella phage SP3]	4.E-15	YP_009804467.1
16	6932	7453	95.95%	173	serine/threonine protein phosphatase [Shigella sonnei]	1.E-121	EHE3968337.1
17	7453	8316	97.91%	287	putative serine/threonine protein phosphatase [Salmonella phage L6jm]	0.0	YP_009856432.1
18	8319	8564	98.77%	81	hypothetical protein [Salmonella enterica subsp. enterica serovar Derby]	1.E-50	ECJ4201699.1
19	8662	8952	100.00%	96	putative thioredoxin [Escherichia virus T5]	1.E-64	YP_006865.1
20	8945	9376	100.00%	143	hypothetical protein SPC35_0037 [Salmonella virus SPC35]	7.E-99	YP_004306520.1
21	9452	9868	100.00%	138	hypothetical protein FG39_gp040 [Escherichia phage vB_EcoS_FFH_1]	5.E-94	YP_009031661.1
22	9946	10359	100.00%	137	lysozyme [Escherichia phage vB_EcoS_AKFV33]	3.E-96	YP_006382340.1
23	10356	11012	100.00%	218	putative holin [Escherichia phage vB_EcoS_FFH_1]	1.E-158	YP_009031663.1
24	11184	11768	99.48%	194	ATP-dependent Clp protease proteolytic subunit [Salmonella phage SP01]	2.E-144	YP_009792580.1
25	11781	12533	100.00%	250	deoxynucleoside-5'-monophosphate kinase [Salmonella virus SPC35]	0.0	YP_004306525.1
26	12533	1266	100.00%	117	membrane lipoprotein [Shigella phage SSP1]	7.E-24	YP_009794526.1

		7					
27	12817	13266	98.66%	149	hypothetical protein HOT63_gp118 [Salmonella phage S131]	1.E-103	YP_009805538.1
28	13223	13921	99.57%	232	hypothetical protein A318_gp047 [Escherichia phage vB_EcoS_AKFV33]	6.E-172	YP_006382346.1
29	14076	14420	95.61%	114	hypothetical protein [Salmonella enterica subsp. enterica serovar Bareilly]	2.E-75	EBS4928293.1
30	14531	14815	100.00%	94	hypothetical protein [Salmonella phage Stp1]	3.E-61	ARQ96238.1
31	14812	15108	98.98%	98	hypothetical protein HOS12_gp116 [Salmonella phage SP01]	1.E-65	YP_009792574.1
32	15474	15773	100.00%	99	hypothetical protein HOS12_gp114 [Salmonella phage SP01]	5.E-65	YP_009792572.1
33	15766	16047	98.92%	93	hypothetical protein HOT55_gp054 [Salmonella phage SP3]	5.E-60	YP_009804485.1
34	16124	16471	100.00%	115	hypothetical protein HOR05_gp139 [Escherichia phage phiAPCEc03]	3.E-78	YP_009785205.1
35	16593	16880	92.08%	101	hypothetical protein HWD12_gp147 [Phage NBSal003]	5.E-62	YP_009857107.1
36	16962	17330	100.00%	138	pyruvate formate-lyase [Salmonella phage Th1]	6.E-84	YP_009849735.1
37	17605	17811	92.65%	68	hypothetical protein SLUR09_00165 [Escherichia phage slur09]	4.E-38	YP_009202192.1
38	17811	18104	98.97%	97	hypothetical protein HWC42_gp062 [Salmonella phage SE11]	1.E-63	YP_009848676.1
39	18172	18339	100.00%	55	hypothetical protein SLUR09_00163 [Escherichia phage slur09]	6.E-31	YP_009202190.1
40	18449	18613	100.00%	54	hypothetical protein A318_gp158 [Escherichia phage vB_EcoS_AKFV33]	3.E-29	YP_006382359.1
41	18606	18857	100.00%	83	hypothetical protein SPC35_0057 [Salmonella virus SPC35]	1.E-54	YP_004306540.1
42	19125	19361	100.00%	78	hypothetical protein HOT55_gp004 [Salmonella phage SP3]	5.E-48	YP_009804435.1
43	19354	19551	100.00%	65	hypothetical protein HWC42_gp057 [Salmonella phage SE11]	8.E-39	YP_009848671.1
44	20207	20791	99.48%	194	HNH endonuclease [Salmonella phage bux]	2.E-139	QIQ61635.1
45	21337	21504	100.00%	55	hypothetical protein HWC51_gp053 [Salmonella phage Th1]	4.E-32	YP_009849743.1
46	21694	21891	100.00%	65	hypothetical protein HOS34_gp010 [Shigella phage SSP1]	2.E-39	YP_009794677.1
47	22345	22692	99.13%	115	hypothetical protein HWC42_gp053 [Salmonella phage SE11]	2.E-76	YP_009848667.1
48	22796	2295	98.08%	52	hypothetical protein HOS12_gp103 [Salmonella phage SP01]	1.E-25	YP_009792561.1

		4					
49	23058	23243	100.00%	61	hypothetical protein SPC35_0061 [Salmonella virus SPC35]	6.E-33	YP_004306544.1
50	23726	23998	100.00%	90	hypothetical protein SLUR09_00141 [Escherichia phage slur09]	9.E-61	YP_009202180.1
51	24545	24820	100.00%	91	hypothetical protein [Salmonella phage vB_SenS_PHB06]	4.E-59	AVQ09915.1
52	24911	25117	100.00%	68	hypothetical protein [Salmonella phage vB_SenS_PHB06]	3.E-40	AVQ09914.1
53	25110	25277	100.00%	55	hypothetical protein SLUR09_00134 [Escherichia phage slur09]	4.E-32	YP_009202177.1
54	25471	25656	100.00%	61	hypothetical protein A318_gp144 [Escherichia phage vB_EcoS_AKFV33]	4.E-36	YP_006382373.1
55	25666	25854	100.00%	62	hypothetical protein HWD13_gp174 [Phage NBSal005]	4.E-36	YP_009857264.1
56	26068	26586	100.00%	172	hypothetical protein SPC35_0067 [Salmonella virus SPC35]	1.E-124	YP_004306550.1
57	26853	27845	98.18%	330	hypothetical protein HWD08_gp077 [Salmonella phage L6jm]	0.0	YP_009856473.1
58	27867	28283	100.00%	138	hypothetical protein HOT54_gp098 [Salmonella phage LVR16A]	3.E-87	YP_009804365.1
59	28607	29554	100.00%	315	hypothetical protein FG39_gp136 [Escherichia phage vB_EcoS_FFH_1]	0.0	YP_009031698.1
60	30498	30932	99.31%	144	hypothetical protein ACSA001_0720 [Salmonella phage vB_SalS_SA001]	3.E-99	QKE54223.1
61	30932	31102	100.00%	56	hypothetical protein HOR86_gp029 [Escherichia phage OSYSP]	6.E-31	YP_009790952.1
62	31171	31620	100.00%	149	cell wall hydrolase SleB [Escherichia phage vB_EcoS_AKFV33]	8.E-107	YP_006382380.1
63	31626	31943	100.00%	105	hypothetical protein SPC35_0074 [Salmonella virus SPC35]	2.E-71	YP_004306557.1
64	32386	33024	100.00%	212	tail fiber protein [Escherichia phage fp01]	1.E-149	YP_009841416.1
65	33078	33260	100.00%	60	hypothetical protein SPC35_0076 [Salmonella virus SPC35]	7.E-34	YP_004306559.1
66	33331	34032	99.14%	233	metallopeptidase [Salmonella phage SP01]	9.E-175	YP_009792544.1
67	34061	34297	100.00%	78	hypothetical protein BI021_gp017 [Salmonella phage NR01]	8.E-51	YP_009283358.1
68	34339	34554	100.00%	71	hypothetical protein A318_gp131 [Escherichia phage vB_EcoS_AKFV33]	3.E-41	YP_006382386.1
69	34619	35134	99.42%	171	hypothetical protein SLUR09_00115 [Escherichia phage slur09]	5.E-121	YP_009202161.1
70	35210	3565	100.00%	149	Swarming motility protein YbiA [Escherichia phage slur09]	1.E-105	YP_009202160.1

		9					
71	35656	35940	100.00%	94	hypothetical protein SLUR09_00113 [Escherichia phage slur09]	4.E-60	YP_009202159.1
72	36018	36494	99.37%	158	ribonuclease H [Salmonella virus SPC35]	2.E-114	YP_004306565.1
73	36494	36775	100.00%	93	hypothetical protein FG39_gp122 [Escherichia phage vB_EcoS_FFH_1]	1.E-61	YP_009031712.1
74	36869	37708	99.64%	279	thymidylate synthase [Salmonella phage 8sent1748]	0.0	QNI21513.1
75	37708	38241	100.00%	177	dihydrofolate reductase [Salmonella phage vB_SenS_PHB06]	9.E-127	AVQ09891.1
76	38238	39383	100.00%	381	aerobic ribonucleoside diphosphate reductase, small subunit [Escherichia phage vB_EcoS_FFH_1]	0.0	YP_009031715.1
77	39490	41925	100.00%	811	aerobic ribonucleoside diphosphate reductase, large subunit [Salmonella phage vB_SalS_ABTNLsp9]	0.0	QPI13678.1
78	41963	42184	100.00%	73	hypothetical protein A318_gp120 [Escherichia phage vB_EcoS_AKFV33]	7.E-45	YP_006382397.1
79	42186	42938	100.00%	250	phosphate starvation-inducible protein [Escherichia phage vB_EcoS_FFH_1]	0.0	YP_009031719.1
80	43291	45165	100.00%	624	ribonucleotide reductase of class III (anaerobic) large subunit [Salmonella phage vB_Sen_I1]	0.0	QJA17767.1
81	45265	45645	98.41%	126	hypothetical protein HWB87_gp054 [Escherichia phage fp01]	1.E-85	YP_009841433.1
82	45638	45844	98.53%	68	hypothetical protein SLUR09_00102 [Escherichia phage slur09]	5.E-39	YP_009202148.1
83	46027	46863	98.20%	278	SIR2 family NAD-dependent protein deacetylase [Salmonella phage SE11]	0.0	YP_009848629.1
84	46850	47032	100.00%	60	hypothetical protein SLUR09_00098 [Escherichia phage slur09]	2.E-36	YP_009202144.1
85	47019	47525	100.00%	168	hypothetical protein HWB87_gp058 [Escherichia phage fp01]	2.E-121	YP_009841437.1
86	47528	47956	97.89%	142	hypothetical protein vBSTyj51_91 [Salmonella phage vB STyj5-1]	1.E-97	QQV89360.1
87	47966	48358	98.46%	130	hypothetical protein [Salmonella enterica subsp. enterica serovar Derby]	4.E-90	ECJ4201776.1
88	48351	48545	96.88%	65	hypothetical protein [Salmonella enterica]	1.E-34	ECP6930370.1
89	48999	51938	99.59%	979	hypothetical protein HWD13_gp071 [Phage NBSal005]	0.0	YP_009857230.1
90	51922	52155	100.00%	77	hypothetical protein SPC35_0100 [Salmonella virus SPC35]	3.E-48	YP_004306583.1
91	52224	52928	100.00%	234	hypothetical protein A318_gp104 [Escherichia phage vB_EcoS_AKFV33]	2.E-170	YP_006382413.1
92	52921	5317	97.59%	83	Phage protein [Escherichia phage T5_ev219]	5.E-51	VUF55513.1

		2					
93	53276	53686	100.00%	136	D3 protein [Salmonella virus SPC35]	6.E-94	YP_004306586.1
94	53723	54019	100.00%	98	hypothetical protein SPC35_0104 [Salmonella virus SPC35]	3.E-65	YP_004306587.1
95	54070	54378	100.00%	102	hypothetical protein SPC35_0105 [Salmonella virus SPC35]	3.E-69	YP_004306588.1
96	54465	54737	100.00%	90	hypothetical protein [Salmonella phage SP1a]	3.E-59	ATI18466.1
97	54738	55709	99.69%	323	DNA ligase [Salmonella enterica subsp. enterica serovar Bareilly]	0.0	EBS4928354.1
98	55912	56691	98.84%	259	NAD-dependent DNA ligase subunit B [Escherichia phage vB_Eco_mar004NP2]	0.0	YP_009824690.1
99	56684	57451	100.00%	255	hypothetical protein BI021_gp050 [Salmonella phage NR01]	0.0	YP_009283391.1
100	57483	59006	100.00%	507	putative replicative DNA helicase [Escherichia phage vB_EcoS_AKFV33]	0.0	YP_006382421.1
101	59003	59893	100.00%	296	toprim domain-containing protein [Shigella flexneri]	0.0	EFQ1274325.1
102	59956	62523	100.00%	855	DNA polymerase I [Salmonella phage L6jm]	0.0	YP_009856518.1
103	62516	63013	100.00%	165	hypothetical protein SPC35_0113 [Salmonella virus SPC35]	3.E-116	YP_004306596.1
104	63010	64362	100.00%	450	putative ATP-dependent helicase [Escherichia phage vB_Eco_mar004NP2]	0.0	YP_009824683.1
105	64362	64867	100.00%	94	hypothetical protein T5.125 [Escherichia virus T5]	3.E-61	YP_006953.1
106	64860	65633	99.22%	257	D11 protein [Salmonella phage vB_SalS_ABTNLsp9]	0.0	QPI13649.1
107	65671	66147	97.47%	158	hypothetical protein SLUR09_00075 [Escherichia phage slur09]	7.E-108	YP_009202121.1
108	66131	67108	99.38%	325	Calcineurin-like phosphoesterase superfamily domain protein [Escherichia phage slur09]	0.0	YP_009202120.1
109	67089	68927	99.18%	612	hypothetical protein HWD08_gp128 [Salmonella phage L6jm]	0.0	YP_009856524.1
110	68931	69413	100.00%	160	D14 protein [Salmonella virus SPC35]	2.E-115	YP_004306602.1
111	69413	70288	100.00%	291	flap endonuclease [Escherichia phage vB_EcoS_FFH_1]	0.0	YP_009031749.1
112	70285	70731	100.00%	148	deoxyUTP pyrophosphatase [Salmonella phage SP01]	5.E-106	YP_009792498.1
113	70694	70957	91.95%	87	hypothetical protein SLUR09_00069 [Escherichia phage slur09]	7.E-53	YP_009202115.1
114	71048	7121	90.57%	65	hypothetical protein HOT33_gp075 [Escherichia phage Gostya9]	4.E-05	YP_009802280.1

		2					
115	71200	71334	87.88%	276	hypothetical protein SLURMMXVI_140074 [Escherichia phage vB_Eco_SLUR25]	5.E-11	SOE45396.1
116	71479	74136	77.80%	889	L-shaped tail fiber assembly protein [Escherichia phage Gostya9]	0.0	YP_009802281.1
117	74136	74558	100.00%	140	hypothetical protein HOS12_gp037 [Salmonella phage SP01]	3.E-95	YP_009792495.1
118	74563	76665	99.00%	700	tail fiber protein [Phage NBSal005]	0.0	YP_009857205.1
119	76666	79515	99.26%	949	putative tail tip protein [Salmonella phage Stp1]	0.0	ARQ96308.1
120	79512	80126	100.00%	204	hypothetical protein HWD13_gp044 [Phage NBSal005]	2.E-145	YP_009857203.1
121	80236	83916	99.51%	1226	tail length tape-measure protein [Salmonella phage SP01]	0.0	YP_009792491.1
122	84000	84368	100.00%	122	hypothetical protein HWD13_gp041 [Phage NBSal005]	3.E-83	YP_009857200.1
123	84430	84834	99.25%	134	hypothetical protein [Salmonella phage 8sent1748]	9.E-93	QNI21561.1
124	84834	85730	99.66%	298	minor tail protein [Escherichia phage Gostya9]	0.0	YP_009802289.1
125	85735	87126	99.14%	463	tail fibers protein [Salmonella phage SP01]	0.0	YP_009792487.1
126	87153	87638	100.00%	161	hypothetical protein T5.146 [Escherichia virus T5]	1.E-116	YP_006974.1
127	87642	88409	98.82%	255	hypothetical protein HOS12_gp027 [Salmonella phage SP01]	0.0	YP_009792485.1
128	88409	88921	100.00%	170	hypothetical protein SPC35_0135 [Salmonella virus SPC35]	2.E-123	YP_004306618.1
129	88981	90357	100.00%	458	capsid and scaffold protein [Salmonella phage SP01]	0.0	YP_009792483.1
130	90375	91007	100.00%	210	prohead protease [Salmonella phage SP3]	3.E-154	YP_009804578.1
131	91011	91499	98.77%	162	putative tail protein [Salmonella phage SE11]	2.E-110	YP_009848729.1
132	91496	92713	99.75%	405	portal protein [Salmonella phage vB_Sen-E22]	0.0	QJD49403.1
133	92713	93150	100.00%	145	hypothetical protein SLUR09_00051 [Escherichia phage slur09]	2.E-103	YP_009202097.1
134	93265	94581	99.77%	438	Terminase-like family protein [Escherichia phage slur09]	0.0	YP_009202096.1
135	94581	95063	100.00%	160	hypothetical protein FG39_gp061 [Escherichia phage vB_EcoS_FFH_1]	2.E-113	YP_009031773.1
136	95074	9699	95.46%	639	hypothetical protein SLUR09_00048 [Escherichia phage slur09]	0.0	YP_009202094.1

		3					
137	97073	97306	100.00%	77	lytic conversion lipoprotein [Salmonella phage oldekolle]	4.E-50	YP_009857756.1
138	97290	97607	80.81%	104	receptor-blocking tail protein [Salmonella phage STWB21]	2.E-50	QTJ63418.1
139	97683	97838	98.04%	51	hypothetical protein HOS12_gp014 [Salmonella phage SP01]	1.E-24	YP_009792472.1
140	97838	97945	100.00%	35	hypothetical protein SLUR09_00046 [Escherichia phage slur09]	7.E-15	YP_009202092.1
141	97942	98136	100.00%	64	membrane protein [Shigella phage SSP1]	1.E-32	YP_009794577.1
142	98129	98374	96.25%	81	hypothetical protein HWD08_gp158 [Salmonella phage L6jm]	7.E-48	YP_009856554.1
143	98371	98844	98.73%	184	homing endonuclease [Salmonella phage SP01]	1.E-112	YP_009792470.1
144	99018	99752	96.31%	258	deoxynucleoside-5'-monophosphatase [Salmonella virus SPC35]	3.E-179	YP_004306484.1
145	99833	100225	99.23%	130	hypothetical protein [Salmonella phage vB_SalS_ABTNLsp9]	6.E-90	QPI13614.1
146	100293	101963	98.56%	556	hypothetical protein HOS12_gp010 [Salmonella phage SP01]	0.0	YP_009792468.1
147	102061	102258	98.46%	65	hypothetical protein HWD13_gp015 [Phage NBSal005]	2.E-37	YP_009857174.1
148	102316	102723	100.00%	135	A2 protein [Phage NBSal005]	9.E-92	YP_009857173.1
149	102825	103076	98.80%	83	hypothetical protein [Salmonella phage vB_SalS_ABTNLsp9]	7.E-52	QPI13610.1
150	103307	103483			No significant similarity found.		
151	104697	105689	97.88%	330	hypothetical protein [Salmonella enterica subsp. enterica serovar Derby]	0.0	ECJ4201720.1
152	105808	106038	98.68%	76	hypothetical protein HWC42_gp099 [Salmonella phage SE11]	2.E-48	YP_009848713.1
153	106088	106237	95.92%	51	hypothetical protein HWA82_gp012 [Escherichia phage SP15]	2.E-22	YP_009829660.1
154	106240	106452	98.57%	70	hypothetical protein [Salmonella phage SP1a]	4.E-42	ATI18587.1
155	106576	106920	99.12%	114	hypothetical protein [Salmonella phage vB_SalS_ABTNLsp4]	4.E-77	QPI13147.1
